# Evaluating nurse preferences for a novel on-body delivery system vs. manual syringes for large-volume subcutaneous drug administration: a survey study

**DOI:** 10.1080/10717544.2025.2484278

**Published:** 2025-04-03

**Authors:** Mehul Desai, Beth Faiman, Lisa A. Gorski, Ashley Miles, Valentina Sterlin, Nicole Curry

**Affiliations:** a Medical Affairs, Enable Injections, Inc, Cincinnati, Ohio, USA; b Department of Hematology and Medical Oncology, Cleveland Clinic, Cleveland, Ohio, USA; c Ascension at Home, Brentwood, Tennessee, USA; d Medical Oncology Outpatient Infusion Center, Jefferson Health, Philadelphia, Pennsylvania, USA; e Division of Hematology & Medical Oncology, New York University Langone Health, New York City, New York, USA; f Patient Safety, Mayo Clinic Jacksonville, Jacksonville, Florida, USA

**Keywords:** Nurses, subcutaneous, on-body delivery system, hyaluronidase, needlestick injury, large-volume

## Abstract

While nurses report challenges with the manual administration of large-volume subcutaneous drugs, these challenges and potential solutions are not captured in the literature. In this cross-sectional study, 45 nurses with experience administering large-volume subcutaneous biologics completed an 18-item survey about preferences for syringes vs. on-body delivery systems. 100% responded that an on-body delivery system seemed easy to learn and use and preferable to syringes. In a drug delivery scenario including comprehensive administration details and assuming equivalent safety and efficacy, 97.78% preferred the on-body delivery system to a daratumumab/hyaluronidase syringe. In the total sample, this preference was primarily attributed to (1) reduced nurse effort due to hands-free delivery, (2) decreased patient pain due to a thinner needle, (3) elimination of needlestick injuries due to a hidden needle, and (4) increased clinic efficiency due to hands-free delivery. 95.56% felt that the on-body delivery system would improve clinic throughput better than syringes. Nurses reported that an on-body delivery system would be easy to learn and use and would improve clinic efficiency and safety. They underscored the importance of decreasing nurse physical burden, needlestick injuries, and patient needle phobia. Contrary to the assumption that speed is paramount, nurses prioritized reducing effort, enhancing administration safety, and improving patient comfort over injection speed.

## 1. Introduction

In oncology, one of the most notable challenges lies in the administration of large-volume subcutaneous (SC) drugs. These drugs are currently administered via a labor-intensive process involving manual injection using a needle and syringe. This method necessitates the exertion of considerable force by the administering nurse, with the process sometimes extending for as long as eight minutes (U.S. Food and Drug Administration, 2020, U.S. Food and Drug Administration, 2020, U.S. Food and Drug Administration, 2017). Throughout this duration, the nurse maintains direct oversight to ensure the safe and accurate delivery of the medication. Despite the significant time and effort nurses devote to administering these large-volume SC drugs (e.g. daratumumab/hyaluronidase [HYAL], rituximab/HYAL, or pertuzumab/trastuzumab/HYAL), nurse preferences regarding the methods of administration are rarely considered and are severely under-researched.

**Figure 1.**
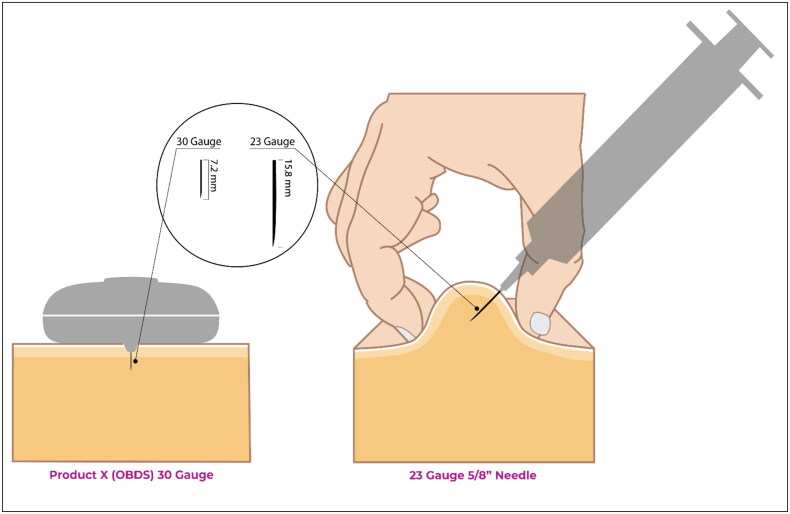
Comparison of the Product X OBDS (30 gauge needle) versus 23 gauge needle. This image was not shown to participants during the study but is included here to illustrate the difference between the size of needles used for OBDSs compared with syringes used in some HYAL-coformulated drugs.

In some healthcare centers, there have been attempts to alleviate the manual administration process by incorporating syringe pumps. However, this approach comes with its own set of challenges: it significantly increases direct administration costs, adds complexity to infusion suite arrangement, requires time-consuming set-up, and often results in crowding and logistical difficulties, further underscoring the need for more efficient and cost-saving alternatives (Ammor *et al.* 2022). While not necessary, large-volume drugs administered with a syringe are sometimes coformulated with HYAL, which increases absorption but necessitates the use of larger needle diameters to reduce delivery time by increasing flow rate (Desai *et al.* 2023). It is important to note that while larger needle diameters can improve delivery speed, they compromise the patient experience because larger needle diameters are associated with increased pain and bleeding (Arendt-Nielsen *et al.* 2006, Jaber *et al.* 2008, Wågø *et al.* 2016). Larger needle diameters can also be more intimidating, potentially increasing patient anxiety, which may require nurses to spend more time counseling patients and can result in missed or delayed injection appointments. By way of example, Figure 1 illustrates a comparison between a 23-gauge needle from a syringe and a 30-gauge needle from an on-body delivery system (OBDS). More nurse- and patient-friendly alternatives for SC drug administration are therefore greatly needed.

OBDSs are a proposed alternative to manually pushed syringes for the administration of large-volume SC drugs; they have the volume capacity of SC syringe-driving pumps, comparable ease of use to autoinjectors, and require minimal preparation before administration. Because OBDSs do not require a manual push and can be built with thinner needles and hidden needle mechanisms, transition to the use of such an OBDS could free up nurse time, decrease physical burden on nurses, improve clinic throughput, reduce or eliminate repetitive strain and needlestick injuries in nurses, and potentially decrease patient pain and needle phobia. While OBDSs are already commonly used in chronic conditions, delivery of monoclonal antibodies and other protein therapeutics is currently in the early stages of development and approval (Desai *et al.* 2023). In-clinic delivery of large-volume SC biologics with an OBDS offers several advantages for administering healthcare providers (HCP) (Philpott 2023), which will be explored in this study.

Although the syringe format allows for rapid administration of drugs relative to intravenous (IV) infusion, it is characterized by several disadvantages, including the potential for repetitive strain and needlestick injuries in nurses and increased patient pain due to needles with larger diameters. Syringes can increase the risk of repetitive strain injuries in nurses who are required to repeatedly apply considerable force over several minutes for several patients per day. Nurses report repetitive strain issues attributed to providing the constant force required for syringe administration of large-volume formulations, and these work-related injuries may require prolonged recovery, particularly for older nurses (Ammor *et al.* 2022). Repetitive strain injuries may also prevent nurses from administering infusions continuously, which can lead to drug misuse and may increase direct and indirect costs (Ammor *et al.* 2022). In a recent meta-analysis of 42 studies including 36,934 nurses, the overall annual prevalence of work-related repetitive strain injuries in nurses was 77.2%, with the highest prevalence of these disorders in the low back (59.5%), neck (53.0%), and shoulder (46.8%) (Sun *et al.* 2023). Work-related repetitive strain injuries can have far-ranging adverse effects and result in high treatment costs, work restrictions, absenteeism, and high turnover for nurses while also negatively impacting patient safety and quality of care (Thinkhamrop *et al.* 2017). Nurses with chronic repetitive strain injuries are more likely to be currently taking medication, using pain medication almost daily, and seeking medical specialist consultations and alternative medications for their injuries (Thinkhamrop *et al.* 2017). Repetitive strain injuries significantly increase direct and indirect costs, with the total cost of diagnostic tests, healthcare, and worker compensation for a nurse with a chronic repetitive strain injury estimated to range from $50,000 to $100,000 (2007 data, not adjusted for inflation) (Gershon *et al.* 2007).

Needlestick injuries are also common among nurses. Prevalence rates of needlestick injuries in nurses vary significantly by region and have been reported in 1.4%–83.9% in the United States (Lee *et al.* 2005). Studies show that half or less than half of needlestick injuries are reported (Rezaei *et al.* 2017), so it is plausible that rates falling in the lower end of the range are underestimates. Costs per injury are considerable, with estimates of $51–$5,000 ($65 million annually) per injury in the United States in 2005 and 2008 (Lee *et al.* 2005). These figures are the most recent available and have not been adjusted for inflation; they are therefore likely to be significantly higher today. These estimates also do not include the costs of lost work time, drug toxicities, emotional distress, litigation, or treatment for long-term complications such as hepatitis or HIV infections, which can add hundreds of thousands of dollars to these costs (Lee *et al.* 2005). In short, needlestick injuries are a common and costly occupational hazard for nurses, and methods for their reduction or elimination would be beneficial on the HCP, healthcare system, and societal level.

The larger needle diameters required for drugs coformulated with HYAL have been shown to increase patient pain (Arendt-Nielsen *et al.* 2006, Jaber *et al.* 2008, Wågø *et al.* 2016) and the pressure required to depress a syringe plunger (Watt *et al.* 2019), thereby increasing the risk of repetitive strain injuries in nurses. Injection-related pain has also been shown to increase pre-administration anxiety, unwillingness or inability to self-administer a medication, and patient adherence (Usach *et al.* 2019). In contrast, research indicates that thinner needles decrease injection-related pain (Desai *et al.* 2023, Wågø *et al.* 2016). A recent clinical study indicated that in addition to ease of use, mobility during infusion, and decreased set-up time, patients may prefer an OBDS over a syringe pump for SC administration due to decreased injection site pain (Wasserman *et al.* 2022). Needle phobia, which has been reported in the majority of the general adult population, may also be alleviated by the thinner needles and hidden needle mechanisms possible with OBDSs (Alsbrooks and Hoerauf, 2022). However, direct comparisons between manually pushed syringes and OBDSs for the administration of large-volume SC drugs regarding their impact on patient pain or needle phobia have not been published and are needed.

Compared with IV infusions, SC preparation and administration improves clinic efficiency by increasing throughput (i.e. decreasing patient chair time) and reduces costs associated with HCP time and resource use (Bittner *et al.* 2018). Further clinic efficiency improvements and cost reductions may be possible with the transition from SC administration in the clinic to home administration (HCP or self-administered). In one Canadian study, compared with IV treatment, self-administered SC immunoglobulin G reduced average administration costs per patient-year by $5386 CDN ($3970 USD; $5931 CDN or $4371 USD in adults, $3177 CDN or $2341 USD in children) with an estimated $31 million CDN ($22.8 million USD) in cost savings for the healthcare system if 80% of patients were switched from clinic-administered IV to self-administered SC treatment during the 7-year observation period (adjusted for inflation to 2020 values) (Ritchie *et al.* 2022). Adjusted for inflation to 2024, SC self-administration reduced average administration costs per patient-year by $6575 CDN ($7240 CDN in adults, $3877 in children) with an estimated $46 million CDN in cost savings for the healthcare system given that 80% of patients were switched from IV to self-administered SC treatment. Self-administered SC treatment has been embraced for several chronic conditions, including diabetes, rheumatoid arthritis, multiple sclerosis, and primary immunodeficiency (Kirkegaard *et al.* 2022), but self-administration of oncology medications remains contentious (Campling and Calman, 2018) due to the tolerability profile of oncology treatments and a lack of reimbursement by Medicare despite the fact that many treatment centers are at or beyond capacity (Desai *et al.* 2024). For example, in the face of staff shortages, insufficient infrastructure to support staff training, inadequate physical space, limited support facilities, run-down buildings, unavailability of reconstituted anti-cancer therapies from commercial manufacturers, and increased demands for and complexity of treatment, National Health Service (NHS) cancer centers in the UK are facing a critical lack of capacity to deliver systemic anti-cancer therapies (Dodhia *et al.* 2023). This lack of capacity forces already overextended HCPs to make impossible decisions, such as refusing access to approved treatments and prioritizing treating some patients at the expense of others (Dodhia *et al.* 2023), and has increased wait times to an unprecedented extent (Price *et al.* 2023). Delays in treatment as seemingly minor as 4 weeks increase mortality 6%-13% in patients with solid cancers, with further increases in mortality seen with additional delays in treatment (Hanna *et al.* 2020).

Therefore, facilitation of self-administration of cancer treatment could provide substantial value. One recent UK pilot study with 14 patients involving the implementation of an educational program paired with home delivery of prefilled syringes of trastuzumab for the treatment of HER2-positive breast cancer found that of the 11 patients who reached the self-administration stage, 100% reported feeling ‘very satisfied’ and 91% ‘very confident’ about self-administration, and 82% reported that it was ‘very easy’ to self-administer cancer treatment (Ng *et al.* 2023). Moreover, total hospital visits were reduced by a mean of 8 appointments per patient (or 10 hours per patient), and 100% reported that reducing hospital visits improved their quality of life (Ng *et al.* 2023). Efforts such as these may alleviate strain on over-burdened healthcare systems and providers.

Unlike the syringes currently used for the administration of large-volume SC drugs, OBDSs use thinner needles, do not require coformulation with HYAL, and feature a hidden needle mechanism. Therefore, OBDSs present a solution to several of the challenges traditionally associated with the SC delivery of large-volume drugs with manually pushed syringes. Hidden needle mechanisms may help alleviate patient needle phobia, while their thinner needles may improve adherence and reduce pain (Alsbrooks and Hoerauf 2022). It is also possible that OBDSs may reduce the physical burden on nurses and the risk of repetitive strain injuries while also increasing clinic throughput (Desai *et al.* 2023).

**Figure 2.**
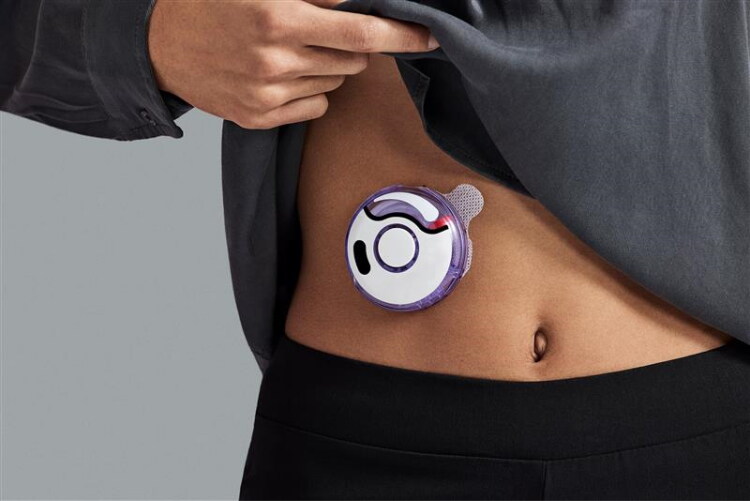
Product X on-body delivery system (enFuse).

To address the shortcomings of syringes and SC syringe pumps, an OBDS, enFuse^®^ (Figure 2, henceforth referred to as Product X), was developed for the delivery of large-volume SC drugs. Unlike SC syringe pumps, Product X is a drug-device combination product in the United States that goes through clinical trials then gains approval for use with each specific drug. To date, it has been FDA-approved for the self-administration of pegcetacoplan (EMPAVELI^®^) for the treatment of paroxysmal nocturnal hemoglobinuria (Philpott 2023).

Despite the burden on nurses associated with the use of manually pushed needle and syringe systems, there is no research available regarding their preferences for potential solutions such as OBDSs. The objective of this study was therefore to evaluate nurses’ preferences for administering viscous, large-volume SC drugs using a manually pushed, prefilled syringe, which necessitates coformulation with HYAL and larger needle diameters, compared with an OBDS (Product X) that allows for hands-free delivery with a thinner needle, hidden needle mechanism, and no need for coformulation with HYAL.

## 2. Materials and methods

### 2.1. Design

This was a cross-sectional, online survey study.

### 2.2. Study setting and sampling

The survey was administered by a third-party vendor with a database of more than 1.5 million respondents in the United States, including nurses. All US nurses in this database were sent an online survey to complete between 29 August and 10 October 2023. The full list of survey questions can be found in Supplementary Information.

### 2.3. Inclusion and exclusion criteria

For the first 13 respondents in this study, the inclusion criteria were academic or private oncology nurse practice in the US and direct experience administering SC daratumumab/HYAL, rituximab/HYAL, and/or pertuzumab/trastuzumab/HYAL. Exclusion criteria included (1) specialization in rheumatology, dermatology, or neurology; (2) retired employment status; (3) no or very little familiarity with the syringe administration of daratumumab/HYAL for the treatment of multiple myeloma administered via a 5-minute manual push by a nurse; (4) no or very little familiarity with the syringe administration of SC rituximab/HYAL for the treatment of chronic lymphocytic leukemia, diffuse large B-cell lymphoma, or follicular lymphoma administered with a 5–7-minute manual syringe push by a nurse; and (5) no or very little familiarity with the syringe administration of SC pertuzumab/trastuzumab/HYAL for the treatment of HER2-positive breast cancer with a 5–8-minute manual syringe push by a nurse. After the first 13 nurses with experience administering daratumumab/HYAL were recruited, these criteria were lifted to allow inclusion of nurses without direct experience administering daratumumab/HYAL.

### 2.4. Data collection

Following completion of the screening questions, nurses were given a written introduction to the survey and shown a 30-second, unbranded demonstration video of an OBDS (Product X) followed by a 4-minute presentation of how Product X works. Once the introduction video and presentation were viewed, nurses completed the survey. Responses from nurses who reported having experience administering daratumumab/HYAL, rituximab/HYAL, and/or pertuzumab/trastuzumab/HYAL using a SC syringe in a set of screening questions were collected, and additional screening questions were used to further divide respondents into subgroups of (1) hematology and oncology nurses and (2) nurses with experience administering daratumumab/HYAL specifically.

Questions in the 18-item survey included demographics (geographic location; type of nursing facility; nursing specialty; sex; age; years of experience in a clinical setting); familiarity with syringe administration of daratumumab/HYAL, rituximab/HYAL, and/or pertuzumab/trastuzumab/HYAL; monthly frequency of personally administering daratumumab/HYAL, rituximab/HYAL, and/or pertuzumab/trastuzumab/HYAL; involvement in the review and evaluation of policies regarding infusion center efficiency; and questions about preferences for an OBDS (Product X) versus a manually pushed, prefilled syringe for SC administration. The full list of survey questions can be found in Supplementary Information.

### 2.5. Data analysis

Responses to survey questions were tabulated and basic descriptive statistics were calculated from the survey data. Results for questions with continuous responses are presented as median (interquartile range [IQR]), results for questions with Likert scale response options are presented as median (IQR) or as number and percentage of participants (*n* [%]) choosing each category, and results for questions with binary responses are presented as number and percentage of participants (n [%]). No formal hypothesis testing was performed.

### 2.6. Ethical considerations

Participants were asked to consent twice during the online study process. When the participant was onboarded online within the third party survey provider network, they were required to agree to the terms and conditions set forth within the agreement necessary for them to become approved to complete surveys. The survey vendor also verified the identity of all participants. Finally, participants reviewed and consented to a Compliance Statement before being given access to the survey for completion.

## 3. Results

### 3.1. Characteristics of the sample

Participant characteristics are summarized in Table 1. The full sample included 45 nurses (86.67% female, median age 41 years [IQR 19], median years of practice 12 [IQR 12]) in the United States (6 in Illinois; 5 in Georgia; 4 in North Carolina; 3 each in California, Maryland, and Massachusetts; 2 each in Colorado, Florida, Minnesota, New York, and Ohio; and 1 each in Arizona, Kentucky, Michigan, Missouri, New Hampshire, New Jersey, Pennsylvania, South Carolina, Texas, Utah, and Virginia) with experience administering daratumumab/HYAL, rituximab/HYAL, and/or pertuzumab/trastuzumab/HYAL. The sample included a subgroup of 18 hematology and oncology nurses (83.33% female, median age 38 years [IQR 14], median years of practice 10 [IQR 10]) and a subgroup of 22 nurses with experience administering daratumumab/HYAL (86.36% female, median age 39.5 years [IQR 12.5], median years of practice 11.5 [IQR 10]). On a scale from 1 (not involved) to 5 (very involved) of involvement in reviewing and evaluating policies about infusion center efficiency, nurses rated themselves a median of 2 (IQR 3.5) in the full sample, 5 (IQR 1) in hematology and oncology nurses, and 4 (IQR 2) in nurses with experience administering daratumumab/HYAL.

**Table 1.** Participant characteristics.

Characteristic	All nurses (*n* = 45)	Hematology and oncology nurses (*n* = 18)	Nurses with experience administering daratumumab/ HYAL (*n* = 22)
Age (years), median (IQR)	41 (19)	38 (14)	39.5 (12.5)
Female	39 (86.67)	15 (83.33)	19 (86.36)
Years of clinical practice, median (IQR)	12 (12)	10 (10)	11.5 (10)
Clinical setting			
Academic (university-affiliated) medical center	22 (48.89)	10 (55.56)	14 (63.64)
Community (not university-affiliated) hospital or private practice	14 (31.11)	5 (27.78)	6 (27.27)
Other	9 (20.00)	3 (16.67)	2 (9.09)
Title/Role			
Staff Nurse	21 (46.67)	5 (27.78)	7 (31.82)
Nurse Director	7 (15.56)	4 (22.22)	5 (22.73)
Nurse Manager	7 (15.56)	2 (11.11)	4 (18.18)
Nurse Supervisor	2 (4.44)	1 (5.56)	1 (4.55)
Nurse Specialist	3 (6.67)	1 (5.56)	1 (4.55)
Infusion Floor Nurse	5 (11.11)	5 (27.78)	4 (18.18)
Involvement in review and evaluation of infusion center efficiency policies			
Self-rated as involved or very involved	18 (40.00)	15 (83.33)	16 (72.73)
Score on scale of 1 (not involved) to 5 (very involved), median (IQR)	2 (3.5)	5 (1)	4 (2)
Self-rated as moderately familiar, familiar, or very familiar with syringe administration			
Daratumumab/HYAL	22 (48.89)	16 (88.89)	22 (100.00)
Rituximab/HYAL	19 (42.22)	14 (94.44)	17 (77.27)
Pertuzumab/trastuzumab/HYAL	18 (40.00)	13 (72.22)	15 (68.18)
Self-rated as familiar or very familiar with SC syringe pump administration of daratumumab/HYAL	16 (35.56)	13 (72.22)	16 (72.72)
Monthly administrations, median (IQR)			
Daratumumab/HYAL	5 (10)	10 (15)	5 (10)
Rituximab/HYAL	0 (2)	1.5 (5)	0 (4)
Pertuzumab/trastuzumab/HYAL	0 (1.5)	2 (10)	0 (5)

Abbreviations: HYAL, hyaluronidase; IQR, interquartile range; n, number of participants. Data presented as n (%) unless otherwise indicated.

The percentage of nurses reporting being more than moderately familiar or very familiar (4 or 5 on a 5-point scale) with using SC syringe pumps for the administration of daratumumab/HYAL was 35.56% (16/45) among all nurses, 72.22% (13/18) among hematology and oncology nurses, and 72.72% (16/22) among nurses with experience administering daratumumab/HYAL. Median personal administrations per average month in the full sample, hematology and oncology nurses, and nurses with experience administering daratumumab/HYAL were, respectively, 5 (IQR 10), 10 (IQR 15), and 5 (IQR 10) for daratumumab/HYAL; 0 (IQR 2), 1.5 (IQR 5), and 0 (IQR 4) for rituximab/HYAL; and 0 (IQR 1.5), 2 (IQR 10), and 0 (IQR 5) for pertuzumab/trastuzumab/HYAL. In the full sample, there were 21 staff nurses, 7 nurse directors, 7 nurse managers, 5 infusion floor nurses, 3 nurse specialists, and 2 nurse supervisors. Among hematology/oncology nurses, 5 were infusion floor nurses, 5 were staff nurses, 4 were nurse directors, 2 were nurse managers, 1 was a nurse specialist, and 1 was a nurse supervisor. Among nurses with experience administering daratumumab/HYAL, there were 7 staff nurses, 5 nurse managers, 5 nurse directors, 4 infusion floor nurses, 2 nurse specialists, and 1 nurse supervisor. Although nurse supervisors, managers, and directors are less likely to directly administer infusions on a regular basis, they were included in the sample because of their previous clinical and current supervisory experience.

### 5.2. Survey results

#### 5.2.1. General preference and ease of use

100% of the nurses in the full sample and both subgroups felt that Product X appeared easy to use and easy to learn based on the demonstration video shown. All nurses in the full sample and both subgroups preferred Product X to high-resistance, manually pushed syringes in a drug-agnostic scenario.

#### 5.2.2. Comparison with current daratumumab/HYAL syringe

When shown a detailed, drug-specific scenario (Table 2) that included parameters beyond delivery time (including administration method, nurse effort, needle gauge, time, patient mobility, and preparation) and assumed comparable efficacy, safety, and cost, 44/45 (97.78%) of the full sample, 17/18 (94.44%) hematology and oncology nurses, and 21/22 (95.45%) nurses with experience administering daratumumab/HYAL reported that they would prefer Product X to the syringe typically used to administer daratumumab/HYAL for the treatment of multiple myeloma.

**Table 2.** Administration considerations included in detailed scenario regarding daratumumab/HYAL administration.

Administration consideration	Product X (OBDS)	Traditional syringe
Administration method	Hands-free, on-body delivery system	Manually pushed syringe
Nurse effort	Hands-free administration Nurse places the device, presses the button, and moves to the next patient or performs other clinical tasks	Direct patient administration Nurse leans over next to the patient, pinches skin to place the needle, and administers via significant force on the syringe plunger
Needle	30 gauge hidden needle in Product X (smaller, hidden needle)	23-25 gauge exposed needle in syringe (larger, exposed needle)
Time	Approximately 10 minutes with no direct supervision during injection	Approximately 5 minutes with direct supervision during injection
Patient mobility	Light to moderate activities permissible during injection	Immobile
Preparation	Vial and device can be delivered to the floor for the nurse to prepare in front of the patient, which minimizes potential for wasted drug due to patient self-discharges against medical advice or no-shows OR Product X has a single-step, hands-free, needleless preparation	Pharmacy draws up syringe in multistep process with various supplies, then it is delivered to the floor; carries risk of wasted drug if patient self-discharges against medical advice or does not attend appointment

In the total sample, the primary reasons for this preference were (1) reduced nurse administration effort due to hands-free delivery, (2) decreased patient pain due to a thinner needle, (3) elimination of needlestick injuries due to a hidden needle mechanism, and (4) increased clinic efficiency due to hands-free delivery.

In hematology and oncology nurses, the primary reasons for this preference were (1) decreased nurse administration effort due to hands-free delivery, (2) increased clinic efficiency due to hands-free administration, and, in equal percentages, (3) decreased needlestick injuries due to a hidden needle mechanism and decreased patient pain due to a thinner needle, and (4) increased patient mobility and easier drug preparation due to flexibility in the preparation location.

In nurses with experience administering SC daratumumab/HYAL, the primary reasons for this preference were (1) reduced nurse administration effort due to hands-free delivery, (2) increased clinic efficiency due to hands-free delivery, (3) elimination of needlestick injury due to a hidden needle mechanism, and (4) decreased patient pain due to a thinner needle.

#### 5.2.3. Comparison with syringe pump

Nearly all nurses in the full sample (97.78%, 44/45), hematology and oncology nurses (94.44%, 17/18), and nurses with experience administering daratumumab/HYAL (95.45%, 21/22) reported that they would prefer Product X to a SC syringe pump for the preparation and administration of 5–25 mL of drug product.

#### 5.2.4. Factors affecting preference regardless of drug and comparison administration method

**Figure 3.**
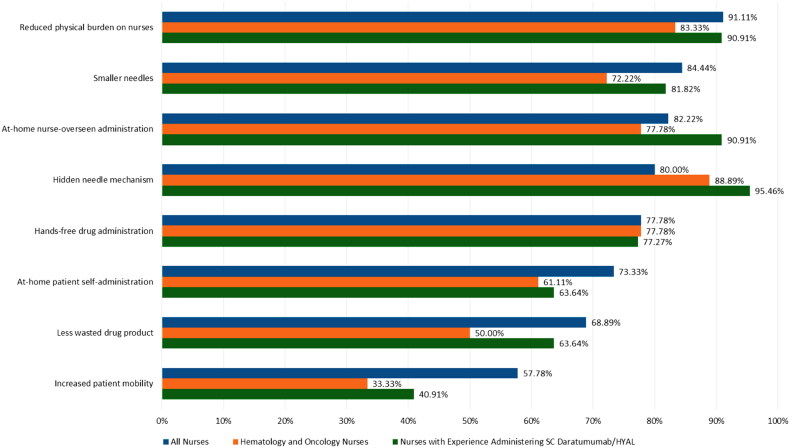
Most important factors in decision-making regarding subcutaneous administration (survey question 9 in supplementary information).

The attributes of Product X most important in nurses’ decision-making regarding large-volume SC administration are summarized in Figure 3, which shows the percentage of nurses in the full sample and in each subgroup who reported each factor to be very or extremely important in their decision-making. As shown, in the full sample, the top three most important features were (1) reduced physical burden on nurses, (2) smaller needles, and (3) the capacity for at-home nurse-overseen administration. In hematology and oncology nurses, the top three most important features were (1) hidden needle mechanism, (2) reduced physical burden on nurses, and (3) hands-free drug administration. In nurses with experience administering daratumumab/HYAL, the top three most important features were (1) hidden needle mechanism, (2) reduced physical burden on nurses, and (3) smaller needles.

Using a 5-point scale (1 = most valuable, 5 = least valuable), median (IQR) scores were 2 (2) in all three groups for hands-free delivery; 2 (3) for all nurses and nurses with experience administering daratumumab/HYAL and 2 (2) for hematology/oncology nurses for flexibility in the site of care; and 3 (2) for all nurses and nurses with experience administering daratumumab and 3 (1) for hematology/oncology nurses for the value of a thinner needle and a hidden needle mechanism.

#### 5.2.5. Clinic efficiency

**Figure 4.**
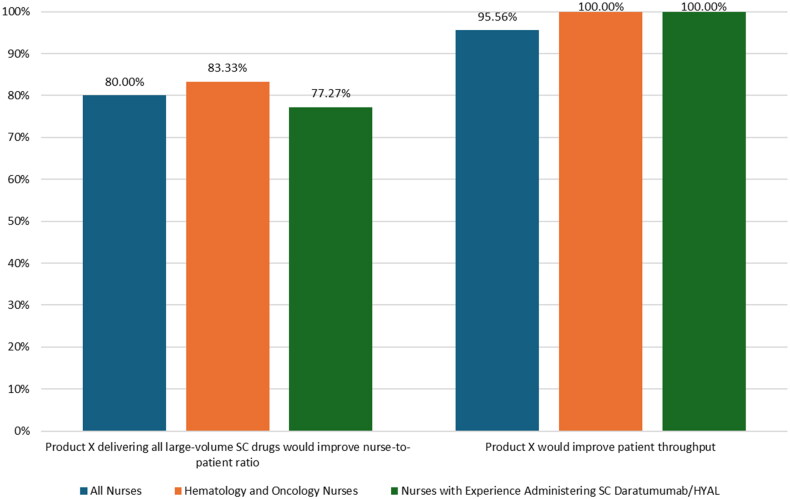
Potential improvements in clinic efficiency with Product X versus syringe (survey questions 10 and 11 in supplementary information).

The results of the survey questions regarding clinic efficiency are shown in Figure 4. The percentage of nurses who felt that Product X being used for the delivery of all large-volume SC drugs in the clinic setting would improve the nurse-to-patient ratio, allowing nurses to manage more patients at a time, was 80.00% (36/45) in the full sample, 83.33% (15/18) in hematology and oncology nurses, and 77.27% (17/22) in nurses with experience administering daratumumab/HYAL. The percentage of nurses who felt that Product X would improve clinic throughput (i.e., decrease patient chair time) was 95.56% (43/45) in the full sample and 100% in hematology and oncology nurses (18/18) and nurses with experience administering daratumumab/HYAL (22/22).

#### 5.2.6. Nurse needlestick injuries

The percentage of nurses who reported that they or a nurse they knew had received a needlestick injury from a syringe was 86.67% (39/45) in the full sample, 83.33% (15/18) in hematology and oncology nurses, and 81.82% (18/22) in nurses with experience administering daratumumab/HYAL. In contrast, the most-reported acceptable range of needlestick injury occurrence in the infusion clinic setting was 1-2% in the full sample and both subgroups, with a weighted mean acceptable prevalence of needlestick injury in nurses of 2.27% reported by all nurses, 2.25% by hematology and oncology nurses, and 1.73% by nurses with experience administering daratumumab/HYAL. The percentage of nurses who believed that a hidden needle would eliminate the risk of needlestick injuries for nurses was 93.33% (42/45) in the full sample, 94.44% (17/18) in hematology and oncology nurses, and 95.46% (21/22) in nurses with experience administering daratumumab/HYAL.

#### 5.2.7. Patient needle phobia

**Figure 5.**
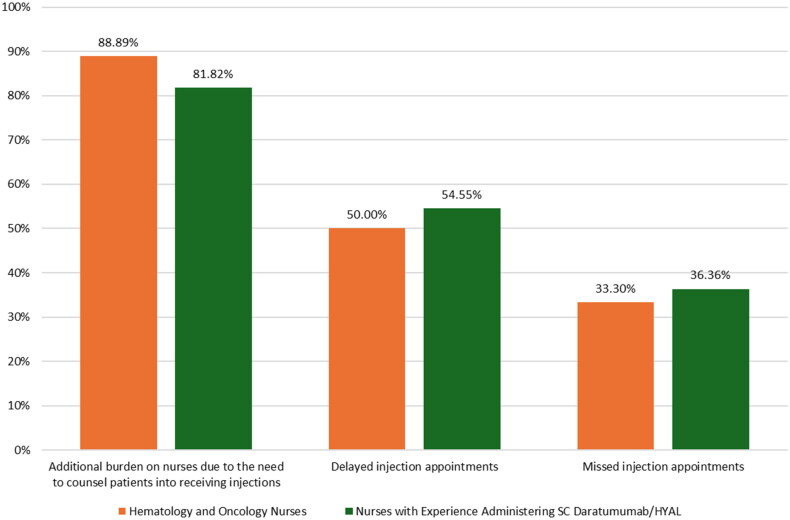
Most common impacts of oncology patient needle phobia on nursing practice (survey question 16 in supplementary information).

Most nurses from the two subgroups reported that the prevalence of needle phobia among oncology patients was 10%–25% (44.44% of hematology and oncology nurses, 50.00% of nurses with experience administering daratumumab/HYAL) or 25%–50% (33.33% of hematology and oncology nurses, 31.82% of nurses with experience administering daratumumab/HYAL), with a weighted mean prevalence of 22.61% reported by all nurses, 23.89% by hematology and oncology nurses, and 23.64% by nurses with experience administering daratumumab/HYAL. The three most commonly reported impacts on nurse practice of needle phobia among oncology patients, shown in Figure 5, were 1) additional burden on nurses due to the need to counsel patients into receiving injections (88.89% [16/18] of hematology and oncology nurses, 81.82% [18/22] of nurses with experience administering daratumumab/HYAL), 2) delayed injection appointments (50.00% [9/18] of hematology and oncology nurses, 54.55% [12/22] of nurses with experience administering daratumumab/HYAL), and 3) missed injection appointments (33.33% [6/18] of hematology and oncology nurses, 36.36% [8/22] of nurses with experience administering daratumumab/HYAL). A total of 94.44% (17/18) of hematology and oncology nurses and 90.91% (20/22) of nurses with experience administering daratumumab/HYAL believed that thinner needles would improve needle phobia for oncology patients.

#### 5.2.8. Patient self-administration

**Figure 6.**
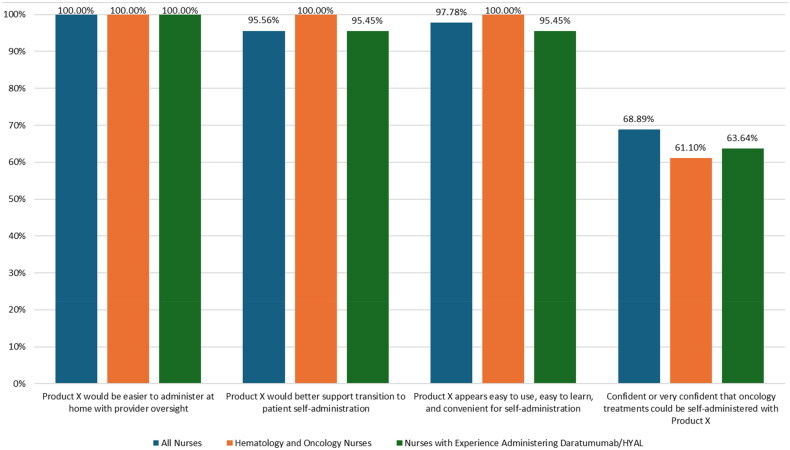
Patient self-administration with Product X versus syringe (survey questions 18-21 in supplementary information).

The results of the survey questions regarding self-administration are shown in Figure 6. Hematology and ­oncology nurses and nurses with experience administering daratumumab/HYAL were divided on whether oncology treatments coformulated with HYAL could be given at home via patient self-administration using high-resistance, manually pushed SC syringes. Among hematology and oncology nurses, 50.00% (9/18) reported being not confident or not confident at all and 50.00% (9/18) reported being confident or moderately confident that oncology treatments could be self-administered at home with current syringes. Among nurses with experience administering daratumumab/HYAL, 59.09% (13/22) reported being not confident or not confident at all and 40.91% (9/22) reported being confident or moderately confident that oncology treatments could be self-administered at home with the current syringes.

In contrast, 100.00% of hematology and oncology nurses and nurses with experience administering daratumumab/HYAL reported that they believed that Product X would be easier to administer than a syringe in the home setting with HCP oversight. The percentage of nurses who reported believing that Product X would better support the transition to patient self-administration than a syringe was 95.56% (43/45) in the full sample, 100% (18/18) in hematology and oncology nurses, and 95.45% (21/22) in nurses with experience administering daratumumab/HYAL. The percentage of nurses who reported that Product X appeared to be easy to use and easy to learn for nurses, as well as convenient for patients self-administering treatment, was 97.78% (44/45) in the full sample, 100% (18/18) in hematology and oncology nurses, and 95.45% (21/22) in nurses with experience administering daratumumab/HYAL. The percentage of nurses who reported being confident or very confident that treatments for oncology could be given via patient self-administration using Product X, assuming the safety profile of the drug allowed for self-administration at home, was 61.11% (11/18) among hematology and oncology nurses and 63.64% (14/22) among nurses with experience administering daratumumab/HYAL.

## 4. Discussion

In this online survey study, 100% of nurses expressed a preference for a novel OBDS (Product X) over traditional manually pushed SC syringe administration of large-volume drugs. Nurses reported that they believed Product X would be easy to learn and easy to use and would improve clinic efficiency, eliminate needlestick injuries, improve patient needle phobia, and improve the ease of and capacity for at-home administration (HCP- or self-administration) of oncology treatments assuming the safety profile of the drug allowed for administration at home. Several findings from this study are potentially surprising. There has traditionally been an emphasis on the speed of large-volume SC delivery, which may compromise both the patient and provider experience by entailing increased patient pain with larger needle diameters, increased physical effort from nurses, and the necessity for direct oversight of administration. In contrast, this survey reveals that nurses place a higher value on aspects other than speed of delivery. In the drug-specific scenario, even though the manually pushed syringe currently used to administer daratumumab/HYAL offers a delivery speed twice as fast, the nurses showed nearly unanimous preference for Product X. It is important to note that hands-free administration with an OBDS frees HCPs to perform other clinical activities, which may provide more economic value despite a longer injection time (Arendt-Nielsen *et al.* 2006, Jaber *et al.* 2008, Wågø *et al.* 2016). Similarly, while some centers are purchasing SC syringe pumps to reduce the physical burden on nurses of manually administering daratumumab/HYAL, the results presented here suggest that nurses would prefer an OBDS over a SC syringe pump. Other methods that centers are using to make the administration easier include the use of butterfly needles as well as larger syringes, such as using a 30 mL syringe to push 15 mL of drug volume instead of using a 15–20 mL syringe. The syringe plunger on a 30 mL syringe may be slightly easier to push than a 15 mL syringe and this can be attributed to the larger plunger surface on the 30 mL syringe. However, the primary source of resistance remains at the needle’s inner diameter (needle gauge), and in combination with the high viscosity of high-concentration drug formulations with hyaluronidase, this has the greatest impact on the overall ease of administration. Consequently, changing the syringe size does not significantly reduce the resistance encountered during drug delivery with large-volume SC drugs formulated with hyaluronidase.

SC treatment dramatically improves clinic efficiency in part by increasing patient throughput (i.e. decreasing patient chair time). For example, while IV administration of trastuzumab takes 30–90 minutes, an SC injection with a manual syringe takes 1–5 minutes (mean 3.3 minutes) (Pivot *et al.* 2012). Other studies have shown that SC administration of trastuzumab with a manual syringe saved a mean of 55 minutes (range 40–81 minutes) of patient chair time and a mean of 17 minutes (range 5–28 minutes) of active HCP time compared with IV infusion (De Cock *et al.* 2016). These time savings translate into cost savings: one prospective observational study conducted in the United Kingdom from 2012 to 2013 found that SC administration cut the cost of HCP time by approximately £100 ($125.76) compared with IV infusion and lowered the cost of consumables by £11.70 ($14.71), for a cost savings per patient over the full course of 18 cycles of treatment of approximately £2,012 ($2,530.31) (Burcombe *et al.* 2013). Again, these figures have not been adjusted for inflation and are likely to be higher today. While these time and cost savings are significant, transition from manually pushed syringes to OBDSs for SC administration of large-volume drugs could offer further savings. Freeing nurses from time spent administering SC treatments is expected to increase clinic efficiency and the capacity to serve more patients.

Although this study did not collect data about repetitive strain injuries in nurses who frequently administer large-volume SC formulations with manually pushed syringes, these injuries are a common occupational hazard faced by nurses and result in significant direct and indirect costs. An OBDS such as Product X could reduce or eliminate the currently high prevalence of nurse repetitive strain and needlestick injuries by reducing or eliminating the use of manually pushed syringes for the delivery of large-volume SC drugs. As reviewed here, nurse repetitive strain and needlestick injuries are associated with significant direct and indirect costs and decreasing the frequency and severity of these injuries could result in substantial cost savings. Importantly, reduced physical burden on nurses and decreased needlestick injuries were the two most valuable factors in decision-making about large-volume SC administration among all nurses as well as both subgroups in this study.

While there is a perception that oncology patients are accustomed to painful treatments and the use of larger needles, which can increase patient pain, the hematology and oncology nurses surveyed here ranked decreased patient pain as the third most important factor in their preference for Product X over the syringe currently used to administer daratumumab/HYAL. These results are echoed by research showing that thinner or hidden needles are associated with decreased patient pain and increased patient adherence even when a longer injection time is required (Arendt-Nielsen *et al.* 2006, Jaber *et al.* 2008, Wågø *et al.* 2016, Philpott, 2023). While Product X can be used with needles slightly thinner than 30 gauge, the recommended needle sizes for the administration of the treatments included in this survey are 23–25 gauge for daratumumab/HYAL (U.S. Food and Drug Administration 2020), 25–30 gauge for rituximab/HYAL (U.S. Food and Drug Administration 2017), and 25–27 for pertuzumab/trastuzumab/HYAL (U.S. Food and Drug Administration 2020). It is important to note that using a higher-gauge (thinner) needle significantly increases the time required for administration.

Although the use of thinner needles may enhance the overall patient experience, this practice is exceedingly rare in clinical settings. The primary deterrent is a significant increase in administration time often deemed unacceptable in crowded, hospital-based infusion centers. Consequently, the largest available needle diameter is used, which reduces delivery time by several minutes at the expense of patient comfort. In this survey, it was shown that needle phobia has several effects on oncology nursing practice. The most significant effect is that nurses end up with more work due to the extra time spent counseling patients to help them feel more comfortable with SC injections. This is paradoxical given that the emphasis has traditionally been placed on the speed of delivery for drugs coformulated with HYAL. A key contributing factor to the speed of HYAL-coformulated drugs is the use of larger needle diameters. Astonishingly, little consideration has been given to potential indirect costs associated with this approach, as underscored by the findings of our survey. The implications are far-reaching. The additional time and effort expended on patient counseling, rescheduling injection appointments, and missed appointments due to the use of needles with larger diameters collectively present substantial and previously overlooked burdens in terms of time-and-motion for infusion centers. The results of this survey echo those of patient surveys about needle phobia, such as the one conducted by Alsbrooks and Hoerauf in a general adult population sample of 2098 participants, 1325 (63.2%) of whom reported needle phobia (Alsbrooks and Hoerauf 2022). This study found that 91.1% of patients believed that thinner needles and 89.7% of patients believed that a hidden needle mechanism would help to alleviate their needle phobia (Alsbrooks and Hoerauf 2022). An OBDS such as Product X could therefore constitute a welcome improvement for patients with needle phobia who nevertheless rely on frequent SC treatments.

Moreover, the use of OBDSs could facilitate home administration (HCP- or self-administration) of hematology and oncology treatments. Self-administered SC treatment has been embraced for several other chronic conditions, including diabetes, rheumatoid arthritis, multiple sclerosis, and primary immunodeficiency. Most SC biotherapeutics used to treat autoimmune diseases can be self-administered by patients at home using an autoinjector, prefilled syringe, or pen injector for low-volume formulations or an OBDS or syringe pump for large-volume formulations. However, while SC administration decreases healthcare costs and burdens (Carrara *et al.* 2021), is easier and quicker than IV infusions (Carrara *et al.* 2021), is safe, well-tolerated, and as efficacious as IV infusion (Usmani *et al.* 2022), and is preferred by HCPs and patients (Bittner *et al.* 2018), self-administration of oncology treatments remains contentious (Campling and Calman 2018).

Subcutaneous backpressure is the resistance encountered during the infusion of substances into the SC tissue layer. While most large-volume SC delivery methods use constant-flow mechanisms that often require manual administration by a HCP and push drugs into the SC space against backpressure, to date there is only one approved large-volume (5–25 mL) OBDS (enFuse) that uses a low-pressure delivery mechanism and allows administration without a manual push by a HCP. Low-pressure delivery with an OBDS adapts the flow rate to changes in SC backpressure, which may lead to a more comfortable experience. Overall, OBDSs are a promising option for the facilitation of comfortable oncology treatment in the home setting.

This study has limitations, including a small sample size, the potential for self-report bias, and a lack of inclusion of patient reports regarding patient-related factors (e.g. pain and needle phobia). As a survey completed by nurses in the United States, these findings may also not generalize to nursing experiences in other countries. However, these findings provide an impetus for further research exploring these potential improvements in the HCP and patient experience with SC drug administration, especially in hematology and oncology.

Further research into the preferences of experienced nurses regarding administration methods for large-volume SC formulations and the potential improvements to both the HCP and patient experience during SC treatment is greatly needed. Moreover, there are gaps in the SC research literature regarding the potential benefits that an OBDS such as Product X could provide for both nurses and patients due to its thinner, hidden needle and the lack of requirement for coformulation with HYAL, as well as the feasibility and benefits of at-home patient self-administration of oncology treatments with an OBDS.

## 5. Conclusion

This study contributes to an awareness of the administration preferences of nurses with experience administering large-volume SC drugs. These findings underscore the significant physical and temporal burden nurses face in administering large-volume formulations by exerting constant force for several minutes per patient, and the alternative presented by an OBDS that allows for hands-free delivery of SC formulations. Nurses in this study felt that the OBDS would be easy to use and easy to learn. Contrary to the assumed primacy of increasing injection speed to the detriment of patient comfort, which has led to some centers purchasing syringe pumps in an effort to increase clinic efficiency, this study demonstrates the greater importance that nurses place on reducing patient pain, alleviating patient needle phobia, decreasing the risk of nurse needlestick injury, and facilitating at-home HCP- or self-administration. Additionally, they expressed that nurses would benefit from the use of an OBDS for the administration of large-volume SC drugs without coformulation with HYAL and the corresponding requirement for larger needle diameters. Nurses in this study felt that an OBDS would improve clinic efficiency and nurse-to-patient ratios. They also reported that the OBDS would eliminate needlestick injuries and provide a better delivery format for at-home HCP- or self-administration than a needle and syringe. Overall, these results indicate that speed of delivery is not the primary factor in nurses’ preferences regarding SC administration when other variables such as nurse effort, preparation, patient mobility, needle size, and needlestick injury risk are considered.

## Supplementary Material

Supplemental Material

## Data Availability

Data used to create this manuscript are available from the corresponding author upon reasonable request.
