# Supplementary material for: Evaluating nurse preferences for a novel on-body delivery system vs. manual syringes for large-volume subcutaneous drug administration: a survey study
Source: Drug Deliv. 2025 Apr 3;32(1):2484278. doi: 10.1080/10717544.2025.2484278 (PMC11980192; doi:10.1080/10717544.2025.2484278)
Supplement: Supplemental Material [file IDRD_A_2484278_SM0985.docx]

**Supplementary Information**

Pre-Interview Survey / Screening Questions

1. Please select your state of work/residence.

1. What is your primary area of specialization?
2. Rheumatology
3. Dermatology
4. Hematology/Oncology
5. Neurology
6. Which of the following best describes your gender?
7. Male
8. Female
9. What is your age?
10. What is the name of the organization for which you currently work? Your organization will be kept anonymous.
11. Which of the following best describes your organization? Please select one.
12. Academic Medical Center (University-affiliated)
13. Community Practice (Private Practice)
14. Other (Please specify): ________________
15. Which of the following best describes your title/role?
16. Staff Nurse
17. Nurse Director
18. Nurse Manager
19. Nurse Supervisor
20. Nurse Specialist
21. Infusion Floor Nurse
22. Home Infusion Nurse
23. Retired
24. How many years have you been practicing in a clinical setting?
25. On a scale of 1-5 with 1 being no familiarity to 5 being very familiar, please rate your familiarity with the syringe administration of Darzalex Faspro (subcutaneous daratumumab/hyaluronidase) used to treat multiple myeloma administered via a 5-minute manual syringe push by a nurse.
26. In an average month, how many times do you personally administer Darzalex Faspro (subcutaneous daratumumab/hyaluronidase)?
27. On a scale of 1-5 with 1 being no familiarity to 5 being very familiar, please rate your familiarity with the syringe administration of Rituxan Hycela (subcutaneous rituximab/hyaluronidase) used to treat Chronic Lymphocytic Leukemia, Diffuse Large B-cell Lymphoma, or Follicular Lymphoma administered via a 5–7 minute manual syringe push by a nurse.
28. In an average month, how many times do you personally administer Rituxan Hycela (rituximab/hyaluronidase)?

1. On a scale of 1-5 with 1 being no familiarity to 5 being very familiar, please rate your familiarity with the syringe administration of Phesgo (subcutaneous pertuzumab/trastuzumab/hyaluronidase) used to treat HER2-positive Breast Cancer via a 5–8 minute manual syringe push by a nurse.
2. In an average month, how many times do you personally administer Phesgo (subcutaneous pertuzumab/trastuzumab/hyaluronidase)?
3. On a scale from 1 to 5, how involved are you in reviewing and evaluating policies around infusion center efficiency? (where 1 = not involved, 3 = moderately involved, and 5 = very involved)

Survey Questions

1. Does the preparation & administration of a drug utilizing Product X appear “easy to use” for nurses based off the video?
2. Yes
3. No
4. Does the preparation & administration of a drug utilizing Product X appear “easy to learn” for nurses based off the video?
5. Yes
6. No
7. Regarding large volume subcutaneous administration, what is your preference for high-resistance, manually pushed syringes such as that used in Darzalex Faspro (subcutaneous daratumumab/hyaluronidase) and Rituxan Hycela (subcutaneous rituximab/hyaluronidase) versus Product X, the On-body Delivery System?
8. Prefer high-resistance, manually pushed syringes
9. Prefer hands-free on-body delivery system
10. From the two profiles shown below, which would you prefer using in the clinic setting for the treatment of multiple myeloma given the following parameters (assuming all other variables like safety and efficacy are comparable):
11. Darzalex Faspro Syringe (Daratumumab/hyaluronidase)
12. Drug X administered with Product X (On-Body Delivery System)

|  | Drug X administered with Product X (OBDS) | Darzalex Faspro Syringe (Daratumumab/hyaluronidase) |
| --- | --- | --- |
| Administration Method | Hands-free on-body deliver system | Manually pushed syringe |
| Nurse Effort | Hands-free administration    Nurse places the device, presses the button and moves to the next patient or performs other clinical tasks | Direct patient administration       Nurse leans over next to the patient, pinches skin to place the needle and administers via significant force on the syringe plunger |
| Needle | 30 gauge hidden needle in Product X (smaller, hidden needle) | 23-25 Gauge Exposed Needle in Syringe (larger, exposed needle) |
| Time | Approximately 10 minutes with no direct supervision during injection | Approximately 5 minutes with direct supervision during injection |
| Patient Mobility | Light to moderate activities permittable during injection | Immobile |
| Preparation | Vial and device can be delivered to the floor for the nurse to prepare in front of the patient which minimizes potential for wasted drug due to patient self-discharges against medical advice or no shows  OR  Product X has a single-step, hands-free, needleless preparation | Pharmacy draws up syringe in multistep process with various supplies, then it is delivered to the floor; carries risk of wasted drug if patient self-discharges against medical advice or does not attend appointment |

1. Regarding your preference from Question 4, please rank the top 3 factors that influenced your decision making (1 being most important, 2 being second most important and 3 being third most important)
2. Nurse effort for administration (hands-free on-body delivery system versus high-resistance manually pushed syringe)
3. Increased clinic efficiency with hands-free administration (longer duration with no direct nurse supervision versus shorter duration with direct nurse supervision)
4. Reduction in needlestick injuries (exposure to needle versus no exposure to needles)
5. Patient Mobility (patient allowed to do light to moderate activities versus being immobile)
6. Patient Pain (smaller needle with Product X versus larger needle with syringe)
7. Drug Preparation (easier preparation process and flexibility in the preparation location)
8. Given the challenge with high-resistance, manually pushed syringes, some centers are utilizing subcutaneous syringe pumps to administer these. On a scale of 1-5 with 1 being no familiarity to 5 being very familiar, what is your level of familiarity with SC syringe pumps?
9. In terms of preparation and administration, when administering 5-25 mL subcutaneously would you prefer Product X over a subcutaneous syringe pump?
10. How would you rank the following 5 features in terms of value to you as a provider? (i.e., top ranked feature #1 is most valuable and #5 is least valuable compared to the rest of the features)

| Product X Features |
| --- |
| Flexibility in the site of care    -In-clinic   -Home infusion under HCP supervision   -Self-administration by patient |
| Hands-free delivery    -No requirement for manual administration by the nurse which may improve nurse to patient ratio and reduce drug administration burden on nurses  -Increased mobility for the patient which provides optionality for hospital protocols to reduce chair time (i.e. fast track injection suites, standing injections) |
| Smaller needle with hidden needle mechanism    -Patient or provider doesn’t see and doesn’t have exposure to needle from beginning to end   -Potential to reduce nurse needle stick injuries and improve patient adherence for those with a fear of needles |
| Dose verification via connected healthcare   -Remote dose verification via the on-body delivery system   -Infusion site reaction monitoring |

1. On a scale from 1 to 5, where 1 = not important at all and 5 = extremely important, how important are the following attributes in your decision-making?
2. Reducing physical burden on nurses (Reducing the use of high-resistance, manually pushed syringes that may cause musculoskeletal issues for nurses)
3. Patient ability to move around and not be tied to an infusion chair during injection – less chair time for the patient via standing injections or implementation of a fast-track injection suite
4. Hands-free drug administration leading to an increased patient throughput in the clinic setting and a more efficient nurse to patient ratio (i.e. 4 or 5 patients to a nurse instead of 3 patients to a nurse).
5. Patient ability to self-administer oncology drugs at home with a device that is easy to use
6. Ability to receive treatment at home via home infusion services with nurse oversight
7. Less wasted drug due to patient no-shows or self-discharges against medical advice – Product X can be delivered to the infusion suite and the nurse can fill it in front of the patient rather than requiring that the drug be pulled into the syringe via pharmacy.
8. Smaller needles (Less pain for patients which may impact adherence)
9. Hidden needle mechanism with potential to reduce needle stick injuries
10. Assume Product X is utilized for the delivery of all large volume SC drugs in the clinic setting (rituximab, daratumumab, trastuzumab, pertuzumab, and others). Given the hands-free administration that doesn’t require direct manual administration by a nurse, would administration with Product X improve the nurse-to-patient ratio so a nurse could manage more patients at a time?
11. Yes
12. No
13. Assume Product X is utilized for the delivery of all large volume SC drugs in the clinic setting (rituximab, daratumumab, trastuzumab, pertuzumab, and others). Given the hands-free administration that doesn’t require direct manual administration by a nurse, would administration with Product X improve patient throughput in the clinic setting?
14. Yes
15. No

The following questions focus on needlestick injuries: 

1. Have you or any nurse you know ever had a needlestick injury from a syringe?
2. Yes
3. No
4. Which of the following ranges sounds reasonable for the occurrence of needlestick injuries to nurses in the infusion clinic setting?
5. Less than 1%
6. 1-2%
7. 3-5%
8. >5%
9. As a reminder the needle in Product X only comes out when the button is pushed, and the needle retracts when the injection is complete so the patient and provider will not see the needle from beginning to end. Would the hidden needle in Product X eliminate the risk for needle stick injuries to nurses?
10. Yes
11. No
12. From your experience, what is the prevalence of needle phobia for patients in the oncology setting?
13. 0-10%
14. 10-25%
15. 25-50%
16. >50%
17. What impact does needle phobia have on your practice? [Select all that apply]
18. Missed injection appointments
19. Delayed injection appointments
20. Additional burden on nurses due to nurse counseling patient into receiving injection
21. Other (free text)
22. Would smaller needles improve needle phobia for patients in the oncology setting?
23. Yes
24. No

Biologic treatments for oncology are mostly administered with HCP oversight in the clinic setting. When administered subcutaneously, several treatments have demonstrated patient preference over intravenous formats. The following questions focus on at-home HCP administration or self-administration in oncology:

1. Assuming the safety profile of the drug allows for self-administration at home, what is your confidence level that treatments for oncology could be given at home via patient self-administration utilizing the current format of high-resistance, manually pushed syringes? (Please rate on a scale of 1 to 5 with 1 being not confident and 5 being very confident)
2. Would Product X be easier to administer in the home infusion setting with HCP oversight than a high-resistance, manually pushed syringe?
3. Yes
4. No
5. Would Product X support the transition to patient self-administration better than a high-resistance, manually pushed syringe?
6. Yes
7. No
8. Does Product X appear easy to use, easy to learn and convenient for patients who are self-administering?
9. Yes
10. No
